# Emotion regulation goals and strategies among individuals with varying levels of sensory processing sensitivity: a latent profile analysis

**DOI:** 10.3389/fpsyg.2024.1364648

**Published:** 2024-04-17

**Authors:** Yiran Liu, Feng Tian

**Affiliations:** ^1^School of Humanities and Social Science, Shanxi Medical University, Taiyuan, China; ^2^Department of Psychiatry, Second Hospital of Shanxi Medical University, Taiyuan, China

**Keywords:** sensory processing sensitivity, emotion regulation, emotion regulation goals, latent profile analysis, China

## Abstract

**Background:**

Emotion regulation (ER) has emerged as a significant factor influencing the well-being of individuals with high sensory processing sensitivity (SPS). However, the interaction between SPS and the underlying mechanisms of ER remains largely unexplored.

**Objective:**

This study aimed to (a) identify profiles of SPS and ER competency using a latent profile analysis (LPA), and (b) investigate the ER goals and strategy use among each profile to better understand ER patterns in highly sensitive individuals with lower ER proficiency.

**Methods:**

A total of 813 Chinese college students (mean age = 21.53 ± 2.48; 74.41% female) completed the Highly Sensitive Person Scale, 16-item Difficulties in Emotion Regulation Scale, Emotion Regulation Goals Scale, Emotion Regulation Questionnaire, and the rumination subscale from the Cognitive Emotion Regulation Questionnaire.

**Results:**

The LPA identified three profiles: “Low SPS - High ER Competency” (41%), “Moderate SPS - ER Competency” (41%), and “High SPS - Low ER Competency” (18%). ER goals varied significantly among these groups. The “High SPS - Low ER Competency” group predominantly pursued contra-hedonic goals and impression management goals, while the “Low SPS - High ER Competency” group focused on pro-hedonic goals. In terms of strategies, the “Low SPS - High ER Competency” group mainly used cognitive reappraisal, the “Moderate SPS - ER Competency” group leaned towards suppression, and the “High SPS - Low ER Competency” group preferred rumination and suppression.

**Conclusion:**

These findings indicate that higher SPS combined with lower ER proficiency is linked to an increased pursuit of contra-hedonic goals and impression management goals, and a reliance on response-focused strategies. This pattern offers new insights for developing psychological support strategies for highly sensitive individuals experiencing mental distress.

## 1 Introduction

Sensory processing sensitivity (SPS), a personality theory that emerged two decades ago, delineates individual differences in terms of their sensitivity to the environment (Greven et al., 2019). Specifically, the heightened SPS reflects four aspects: deeper information processing, enhanced emotional reactivity and empathy, increased sensory sensitivity, and ease of overstimulation (Greven et al., 2019; Pluess et al., 2023). Although SPS is distinct from psychological disorders, individuals with higher SPS often experience amplified emotional distress. This is evidenced by a meta-analysis study involving 5,326 participants, which identified a significant positive correlation between SPS and negative affect, such as anxiety, depression, and stress in both children and adults. Additionally, high SPS was also related to positive affect in children (Lionetti et al., 2019). Although negative affect linked to SPS does not invariably lead to psychopathology, it may predispose individuals to more severe psychological distress, particularly if maladaptive cognitive responses to the affect occur (Wyller et al., 2017). In the context of SPS, emotion regulation (ER) has emerged as the most studied cognitive process.

ER is defined as individuals’ efforts to manage the type, timing, experience and expression of their emotions (Gross, 1998a). While delving into the role of ER within the relation between SPS and psychological distress, researchers have primarily relied on two models – the multidimensional model (Gratz and Roemer, 2004) and the process model (Gross, 1998a). The multidimensional model proposes six dimensions of ER competency, suggesting that deficits in these dimensions could act as transdiagnostic risk factors for psychopathology (Gratz and Roemer, 2004; Cludius et al., 2020). These competencies include awareness and clarity of emotional responses, acceptance of these responses, the ability to control impulsivity and engage in goal-directed behaviors when experiencing distress, and the adaptation of appropriate ER strategies (Gratz and Roemer, 2004). On the other hand, the process model emphasizes ER as a progression through sequential stages: (1) Identification of the incongruence between an individual’s intended emotional state and the currently experienced state; (2) Selection of regulation strategies; (3) Implementation of a particular strategy by employing tactics; (4) Monitoring the effectiveness of other stages in altering one’s emotional experience towards the desired affective state (McRae and Gross, 2020). Variations or difficulties in individuals’ ER often arise from the selection and implementation of ER strategies, with cognitive reappraisal and expressive suppression being the most commonly investigated strategies in empirical studies (John and Eng, 2014). Cognitive reappraisal, considered as an antecedent-focused strategy, involves altering the emotional impact of an event by reinterpreting its meaning. This strategy is employed before the full emotional response unfolds, aiming to change the initial emotional reaction. In contrast, expressive suppression, a response-focused strategy, involves inhibiting emotional expressions. This approach is typically employed after the emotional response has occurred, targeting the control of outward expressions rather than the internal emotional experience (Gross, 1998a,b).

Research exploring the interaction of SPS and ER has predominantly employed the multidimensional model. This approach, as assessed through the subscales of the Difficulties in Emotion Regulation Scale (DERS; Gratz and Roemer, 2004), revealed that individuals with higher level of SPS tended to be more aware of their emotional states, but at the same time faced significant ER challenges. These challenges included nonacceptance of emotional responses, difficulty in engaging into goal-oriented behavior, impulse control issues, and limited access to diverse ER strategies. Notably, some of these challenges further served as mediators between SPS and negative affect (Brindle et al., 2015). Although no significant relationship was observed between emotional clarity and SPS, clues can be found in the studies of alexithymia — a personality dimension characterized by difficulties in identifying, articulating, and communicating emotions (Taylor et al., 1991). These studies have found a positive correlation between SPS and alexithymia, particularly in the aspects of identifying and describing feelings (Liss et al., 2008; Rigby et al., 2020; Jakobson and Rigby, 2021), implying that a lack of emotional clarity could be a relevant issue among individuals with high SPS. Further investigations into factors such as attachment style revealed that the positive correlation between SPS and ER difficulties, as measured by DERS, was consistent across various attachment styles. This indicated that highly sensitive individuals, even with secure attachment, are less capable of ER compared to their less sensitive counterparts (Montoya-Pérez et al., 2021).

Recent studies have extended the understanding of ER within the context of SPS by focusing on specific ER strategies. A longitudinal study found that SPS measured at age 3 was positively correlated with the use of ruminative strategies at age 9. This correlation was especially pronounced in children exposed to permissive parenting, leading to higher levels of depression at ages 9 and 12 (Lionetti et al., 2022). Furthermore, a cross-sectional study found that participants with higher SPS was more likely to adopt dysfunctional attitudes and suppression strategies, and experienced higher levels of depression, anxiety, and stress, as compared to the low SPS population (Eşkisu et al., 2022). These studies collectively point towards a potential preference for response-focused strategies, such as rumination (persistently thinking of negative emotions; Ragen et al., 2016) and suppression, highlighting a nuanced ER characteristic of sensitive individuals.

Despite the previous studies using different models of ER and consistently finding that ER influences the relationship between SPS and mental distress (Brindle et al., 2015; Eşkisu et al., 2022; Lionetti et al., 2022), there remains a notable gap in the empirical research. To date, investigations have predominantly concentrated on the influencing factors and consequences of ER in the context of SPS, rather than delving into the mechanisms of ER. In particular, the motivations driving individuals with different SPS levels to engage in ER are not well-understood. A promising area of study in this context is the exploration of motivations to regulate emotions, recently conceptualized as emotion goals. These goals refer to the cognitive depiction of specific emotional states that individuals aim to attain (Mauss and Tamir, 2014). In the process model of ER, emotion goals are critical as they initiate the entire regulation process, guide the selection of strategies, and influence the outcomes of ER (Eldesouky and Gross, 2019). Tamir’s taxonomy divided these goals into two primary classes: hedonic goals, focusing on immediate emotional state changes, and instrumental goals, aiming at long-term benefits beyond the immediate emotional experience (Tamir, 2016). Building on this taxonomy, the Emotion Regulation Goals Scale (ERGS) was developed to assess five specific goals relevant to daily life (Eldesouky and English, 2019a). These included pro-hedonic goals (the objective to experience positive emotions), contra-hedonic goals (the objective to experience negative emotions), performance goals (the objective to carry out a particular activity), pro-social goals (the objective to affect social interactions or relationships), and a self-focused social goal named impression management (the objective to present oneself in a specific manner to others).

Expanding on the discussion of ER goals, recent studies have examined how these goals associated with various personality traits, further illuminating the complex dynamics of ER (Ford and Tamir, 2014; Eldesouky and English, 2019a). For example, individuals high in Openness often pursue performance goals, reflecting a strong emphasis on achievement. Neurotic people are more inclined to contra-hedonic goals and impression management goals, possibly due to their heightened perception of the world as threatening and increased sensitivity to negative emotions and rejection (Rolland, 2002; George et al., 2011; Eldesouky and English, 2019a). These ER goals significantly influence the choice of ER strategies. Specifically, contra-hedonic goals and impression management goals were linked to a greater reliance on suppressive and ruminative strategies (response-focused strategies), while pro-hedonic goals were linked to more use of antecedent-focused strategies such as cognitive reappraisal (Eldesouky and English, 2019b; Brandão et al., 2023). This indicates a critical role of personality traits in ER, suggesting that personality shapes an individual’s tendency to regulate emotions towards specific aims and that individuals often prefer emotional states that align with their personality traits.

Although SPS has been associated with deficits in ER competencies and a tendency towards response-focused strategy use, the specific impact of SPS as a personality trait on the mechanisms of ER remains largely unexplored. Inspired by studies on ER goals, the present study sought to explore which emotion goals sensitive individuals prefer and how these goals relate to their strategy use for ER.

Due to the interaction between SPS and early experience (Greven et al., 2019), studies have consistently shown that highly sensitive adults exposed to predominantly positive or negative parenting environments exhibited correspondingly more positive or negative affect (e.g., Aron et al., 2005; Liss et al., 2005). It was also posited that individuals with high SPS, when raised in favorable parenting environments, were likely to develop more adaptive ER patterns compared to those raised in less advantageous environments. However, there has been discrepancy regarding the impact of attachment on the relationship between SPS and ER (Montoya-Pérez et al., 2021; Lionetti et al., 2022).

Given that ER capacities are inherently derived from the interplay between individual predispositions and their social experiences (Thompson and Waters, 2020), the current study opted to utilize latent profile analysis (LPA) to identify homogeneous subgroups of participants characterized by similar types of SPS and ER competencies, rather than by their attachment styles. This approach aimed to elucidate the combination of SPS and ER competencies within individuals, specifically targeting those with specific levels of SPS whose ER are less capable, regardless of their early experiences. Furthermore, we expected to identify a subgroup of sensitive individuals who possessed certain well-developed ER competencies. Subsequently, how these subgroups differ in their desired emotional states and ER strategy use would be further investigated.

Previous studies employing latent class analysis to assess SPS have consistently revealed the existence of subgroups characterized by high, moderate, and low levels of SPS (Lionetti et al., 2018; Pluess et al., 2018). Based on these findings, the current study hypothesized the emergence of at least three distinct profiles reflecting a combination of SPS and ER competencies. Each of these profiles was expected to exhibit unique ER habits, highlighting the diverse ways in which SPS interacts with ER.

## 2 Methods

### 2.1 Participants

This present study adopted a voluntary recruitment approach, respondents were 813 Chinese university students. Participants consisted of 208 males and 605 females, aged between 17 and 33 years (*M* = 21.53, SD = 2.48). The majority of the students were undergraduates (71.34%).

### 2.2 Procedures

Participants were recruited through online advertisements on major social media platforms in China. The advertisements provided a brief introduction, inclusion criteria, and potential risks of participation (psychological discomfort). Before participating in the survey, all participants were informed about their right to withdraw at any time and were assured of privacy protection. Informed consent was obtained from each participant. In appreciation of their invaluable contributions, the research team promised to provide participants with detailed explanations of the study’s findings upon its completion. All data were collected using Wen Juan Xing, a secure online survey system.

### 2.3 Measures

#### 2.3.1 Demographic information

Participants provided their demographic information through a self-report questionnaire, which included details such as gender, age, education level, and only-child status.

#### 2.3.2 Sensory processing sensitivity

SPS was assessed using the Highly Sensitive Person Scale (HSPS; Aron and Aron, 1997; Chinese version: Zhang and Zhang, 2023). The Chinese version of the HSPS comprises 27 items, distributed across six factors: emotional reactivity (α = 0.78), low sensory threshold (α = 0.62), ease of excitation (α = 0.76), aesthetic sensitivity (α = 0.65), punishment sensitivity (α = 0.45), and depth of processing (α = 0.62). Respondents rated each item on a 7-point scale ranging from 1 (strongly disagree) to 7 (strongly agree). While the Cronbach’s alpha coefficient for the punishment sensitivity subscale did not reach a satisfactory level (Taber, 2018), mirroring the findings reported by Zhang and Zhang (2023) with a coefficient of 0.46, the issue has been acknowledged and addressed by the authors in their discussion. In this study, the Cronbach’s alpha coefficient for the HSPS was 0.86.

#### 2.3.3 Emotion regulation competency

ER competency was assessed using the 16-item version of the DERS (DERS-16; Bjureberg et al., 2016; Chinese version: Wang et al., 2021). The DERS-16 is comprised of five subscales, each reflecting a specific deficit in ER competency: lack of emotional clarity (α = 0.73), difficulties engaging in goal-directed behavior (α = 0.86), impulse control difficulties (α = 0.83), limited access to effective ER strategies (α = 0.84), and nonacceptance of emotional responses (α = 0.73). Respondents rated each item on a 5-point scale ranging from 1 (almost never) to 5 (almost always). In this study, the overall Cronbach’s alpha coefficient for the DERS-16 was 0.92.

#### 2.3.4 Emotion regulation goals

The ER goals were assessed using the ERGS, which was originally developed by Eldesouky and English (2019a). It comprised 18 items and five subscales corresponding to five ER goals: pro-hedonic goals, contra-hedonic goals, performance goals, pro-social goals, and impression management goals. All items were rated on a 7-point scale where 1 = never, 7 = always.

After obtaining consent from the original authors, the research team adapted the scale using a double translation manner (Beaton et al., 2000; Sousa and Rojjanasrirat, 2011). With the goal of maintaining the original dimensions, confirmatory factor analyses were conducted to assess the scale’s validity. The five-factor model encompassing 18 items indicated suboptimal fit due to a low factor loading for the item “To avoid being distracted by how you are feeling” (0.30) in the performance goals subscale (Meyers et al., 2013). Item response theory also suggested low discrimination for this item (α_1_ = 0.47; Baker and Kim, 2007). Considering similar findings in a previous adaptation study of the ERGS (Brandão et al., 2022), the research team discussed and decided to remove this item. The revised scale presented satisfactory fit indices: χ2/df = 4.66; CFI = 0.93; TLI = 0.91; GFI = 0.93; SRMA = 0.06; RMSEA = 0.07 (Meyers et al., 2013). The Cronbach’s alpha coefficients for each subscale were as follows: pro-hedonic goals (α = 0.75), contra-hedonic goals (α = 0.71), performance goals (α = 0.79), pro-social goals (α = 0.80), and impression management goals (α = 0.81). The Cronbach’s alpha coefficient for the total ERGS was 0.84.

#### 2.3.5 Emotion regulation strategies

Cognitive reappraisal and expressive suppression were assessed using the Emotion Regulation Questionnaire (Gross and John, 2003; Chinese version: Wang et al., 2007). This is a 10-item scale rating on 7-point scale where 1 = strongly disagree, 7 = strongly agree. In the current study, Cronbach’s alpha coefficients were 0.80 for cognitive reappraisal and 0.74 for expressive suppression. Rumination was measured with four items (5-point scale) from the Cognitive Emotion Regulation Questionnaire (Garnefski et al., 2001; Chinese version: Zhu et al., 2007). The Cronbach’s alpha coefficient was 0.73.

### 2.4 Data analysis

All data collected was initially processed and analyzed in R (version 4.2.2) with R Studio. Mplus (version 8.3) was then used to conduct LPAs to discover participants’ SPS - ER competency profiles.

While conducting LPAs, models with 1–5 profiles were sequentially obtained and their fitness was firstly evaluated based on the following criteria: the Akaike Information Criteria (AIC; Akaike, 1974), the Bayesian Information Criterion (BIC; Schwarz, 1978), and the sample-size-adjusted BIC (SABIC; Sclove, 1987). Models with lower values were considered to have better fitness. Another criterion for fitness was entropy, which ranged from 0 to 1. A value close to 1 indicated higher fitness, and a value above 0.80 was considered acceptable (Muthén, 2004). Likelihood tests, including the Lo–Mendell–Rubin likelihood ratio test (LMR) and the bootstrapped likelihood ratio test (BLRT), were further utilized to compare these models. When the difference between the k-profile model and (k-1)- profile model was significant (*p* < 0.05), this suggested that the k-profile model was superior (Magidson and Vermunt, 2002).

After identifying the optimal profile model, demographic variables were assessed as covariates using the three-step approach (R3STEP; Asparouhov and Muthén, 2014). This approach accounted for the possibility of misclassifying latent classes caused by the introduction of predictive covariates. Specifically, the covariates included gender (0 = female, 1 = male), age (1 = 17–20 years old, 2 = 21–25 years old, 3 = above 25 years old), and only-child status (0 = only-child, 1 = non-only child). The selection of these covariates was informed by prior studies indicating differences in SPS and ER across these variables (Lionetti, 2020; Wang et al., 2021; Zhang and Zhang, 2023). ER goals and ER strategies, regarded as distal outcomes, were further assessed using the BCH method (Lanza et al., 2013), enabling the examination of how the identified profiles differ in terms of ER habits.

## 3 Results

### 3.1 Descriptive analysis

Table 1 displays the means, standard deviations, and intercorrelations among major variables. Regarding SPS, it was positively correlated with all subscales of the DERS. SPS also showed significant positive correlations with most ER goals, except for the pro-hedonic goals. Among the three ER strategies, rumination showed the strongest positive correlation with SPS.

**Table 1:** Descriptive analysis.

	1	2	3	4	5	6	7	8	9	10	11	12	13	14
1. SPS	–													
2. IMPULSE	0.39^***^	–												
3. NONACCEPTANCE	0.44^***^	0.49^***^	–											
4. CLARITY	0.18^***^	0.42^***^	0.42^***^	–										
5. GOALS	0.48^***^	0.619^**^	0.49^***^	0.31^***^	–									
6. STRATEGIES	0.46^***^	0.63^***^	0.65^***^	0.48^***^	0.60^***^	–								
7. Pro-hedonic	0.02	−0.08^*^	−0.11^**^	−0.23^***^	−0.07^*^	−0.29^***^	–							
8. Contra-hedonic	0.19^***^	0.20^**^	0.22^***^	0.27^**^	0.16^***^	0.38^***^	−0.38^***^	–						
9. Performance	0.10^**^	−0.04	−0.07	−0.14^***^	−0.05	−0.10^**^	0.34^***^	−0.14^***^	–					
10. Pro-social	0.20^***^	0.05	0.19^***^	−0.01	0.12^**^	0.03	0.42^***^	0.01	0.20^***^	–				
11. Impression management	0.24^***^	0.10^**^	0.26^***^	0.06	0.16^***^	0.15^***^	0.28^***^	0.06	0.18^***^	0.74^***^	–			
12. Reappraisal	0.07*	−0.16^***^	−0.10^**^	−0.16^***^	−0.09^**^	−0.27^***^	0.49^***^	−0.25^***^	0.18^***^	0.29^***^	0.18^***^	–		
13. Suppression	0.14^***^	−0.02	0.20^***^	0.25^***^	0.03	0.17^***^	−0.23^***^	0.23^***^	−0.02	−0.10^**^	0.03	−0.04	–	
14. Rumination	0.47^***^	0.39^***^	0.47^***^	0.26^***^	0.42^***^	0.51^***^	−0.04	0.20^***^	0.00	0.21^***^	0.22^***^	−0.06	0.06	–
Mean	5.01	2.32	2.43	2.47	2.92	2.66	4.95	2.90	5.30	4.41	4.77	5.18	3.97	3.46
SD	0.70	1.02	1.02	1.08	1.09	1.02	1.21	1.21	1.13	1.15	1.20	1.00	1.33	0.74

SPS, Sensory processing sensitivity; NONACCEPATNCE, Nonacceptance of emotional responses; CLARITY, Lack of emotional clarity; GOALS, Difficulties engaging in goal-directed behavior; STRATEGIES, Limited access to effective ER strategies. ^*^p < 0.05; ^**^p < 0.01; ^***^p < 0.001.

Regarding hedonic goals, distinct correlation patterns were observed. Pro-hedonic goals were positively correlated with cognitive reappraisal, but negatively correlated with expressive suppression and with all subscales of the DERS. In contrast, contra-hedonic goals displayed positive correlations with expressive suppression, rumination, and all DERS subscales, while showing a negative correlation with cognitive reappraisal.

### 3.2 Latent profile analysis

Table 2 presents the fit statistics for models 1–5 derived from the LPAs. A trend of decreasing AIC, BIC, and SA-BIC values was observed with an increasing number of profiles across these models. Despite the 4-profile model showing the highest entropy, its LMR test result was not significant. Consequently, the 3-profile model was identified as the most optimal, evidenced by a satisfactory entropy value (0.82) and statistically significant LMR and BLRT results (*p* < 0.05).

**Table 2:** Fit statistics of latent profiles analysis.

Model	AIC	BIC	SA-BIC	Entropy	LMR *p*	BLRT *p*	Latent class proportion
1 Class	13642.573	13698.982	13660.875	n/a	n/a	n/a	n/a
2 Class	12197.842	12287.155	12226.819	0.84	<0.001	<0.001	0.57/0.43
3 Class	11808.333	11930.552	11847.986	0.82	0.026	<0.001	0.41/0.41/0.18
4 Class	11727.172	11882.296	11777.501	0.85	0.252	<0.001	0.42/0.04/0.39/0.15
5 Class	11648.624	11836.654	11709.630	0.78	0.168	<0.001	0.33/0.31/0.05/0.20/0.11

**Figure 1:**
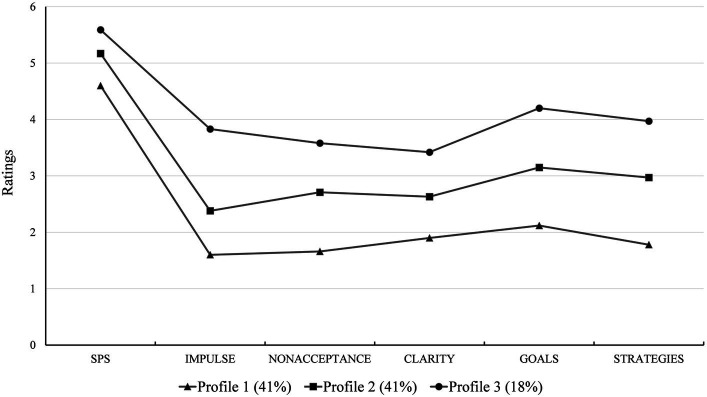
SPS - ER competency profiles.

In the selected 3-profile model, the first profile comprised participants displaying low levels of SPS scores and deficits in all five ER competencies, hence named the “Low SPS - High ER Competency” group. The second profile, termed the “Moderate SPS - ER Competency” group, consisted of participants with medium levels of both SPS and ER competency scores. The first profile and the second profile each represented 41% of the total sample. The third profile, accounting for 18% of the participants, was identified as the “High SPS - Low ER Competency” group, characterized by a higher SPS level and significant deficits in all ER competencies (see Figure 1).

### 3.3 Demographic covariates of latent profile

Table 3 details the outcomes of the multinomial logistic regression analyses conducted to explore the demographic covariates of the SPS - ER competency profiles. The results indicated that gender and age were significant differentiating factors among these profiles. Specifically, compared to the “Low SPS - High ER Competency” group, male participants were less likely to be classified into the “Moderate SPS - ER Competency” group (OR = 0.62, *p* < 0.05) and the “High SPS - Low ER Competency” group (OR = 0.48, *p* < 0.05). However, the direct comparison between the “Moderate SPS - ER Competency” group and the “High SPS - Low ER Competency” group showed no significant gender-based differences in the classification likelihood. The analysis of age differences across these profiles revealed that younger participants were more likely to fall into the “High SPS-Low ER Competency” group, while there were no significant age-related differences between the “Moderate SPS - ER Competency” group and the “Low SPS - High ER Competency” group (OR = 0.91, *p* > 0.05).

**Table 3:** Covariates of the latent profiles.

	Female gender	Only-child	Age
Low SPS - High ER Competency	234 (69%)	158 (46%)	M = 1.73, SD = 0.62
Moderate SPS - ER Competency	254 (77%)	141 (43%)	M = 1.69, SD = 0.58
High SPS - Low ER Competency	116 (81%)	70 (49%)	M = 1.57, SD = 0.56
*Moderate SPS - ER Competency* versus *Low SPS - High ER Competency*			
B	−0.48	0.18	−0.10
OR	0.62	1.20	0.91
*p*	0.003	0.367	0.494
*High SPS - Low ER Competency* versus *Low SPS - High ER Competency*			
B	−0.73	−0.09	−0.50
OR	0.48	0.91	0.61
*p*	0.000	0.645	0.000
*High SPS - Low ER Competency* versus *Moderate SPS - ER Competency*			
B	−0.25	−0.28	−0.40
OR	0.78	0.76	0.67
*p*	0.342	0.175	0.012

M, Mean; SD, Standard deviation; OR, Odds ratio.

### 3.4 Distal outcomes of latent profile

Chi-square analyses were conducted to assess differences in ER habits among the three profiles. As shown in Table 4, these profiles demonstrated a range of significant distinctions in all of the ER goals and strategies. In terms of ER goals, the “High SPS - Low ER Competency” group exhibited distinct preferences compared to the “Low SPS - High ER Competency” group across four goals. The “High SPS - Low ER competency” group was more inclined towards contra-hedonic goals, pro-social goals, and impression management goals, whereas the “Low SPS - High ER competency” group demonstrated a greater preference to pro-hedonic goals. Additionally, the “High SPS - Low ER Competency” group differed from the “Moderate SPS - ER Competency” group by showing a greater pursuit of contra-hedonic goals and impression management goals. The “Moderate SPS - ER Competency” group was distinguished from the “Low SPS - High ER Competency” group across four ER goals, showing lesser focus on pro-hedonic goals and performance goals but a higher tendency towards contra-hedonic goals and impression management goals.

**Table 4:** Distal outcomes of the latent profiles.

	P1	P2	P3	Pairwise comparisons		
Outcome	Mean (SE)	Mean (SE)	Mean (SE)	P1 versus P2 versus P3		
Pro-hedonic	5.27 (0.07)	4.72 (0.07)	4.73 (0.13)	P1 versus P2 = ^***^	P1 versus P3 = ^***^	P2 versus P3 = ns
Contra-hedonic	2.47 (0.06)	3.09 (0.08)	3.47 (0.13)	P1 versus P2 = ^***^	P1 versus P3 = ^***^	P2 versus P3 = ^*^
Pro-social	4.30 (0.07)	4.42 (0.07)	4.67 (0.11)	P1 versus P2 = ns	P1 versus P3 = ^*^	P2 versus P3 = ns
Performance	5.41 (0.06)	5.21 (0.07)	5.26 (0.11)	P1 versus P2 = ^*^	P1 versus P3 = ns	P2 versus P3 = ns
Impression management	4.48 (0.07)	4.88 (0.07)	5.19 (0.12)	P1 versus P2 = ^***^	P1 versus P3 = ^***^	P2 versus P3 = ^*^
Reappraisal	5.37 (0.05)	5.13 (0.06)	4.86 (0.11)	P1 versus P2 = ^**^	P1 versus P3 = ^***^	P2 versus P3 = ^*^
Suppression	3.68 (0.08)	4.19 (0.07)	4.15 (0.13)	P1 versus P2 = ^***^	P1 versus P3 = ^**^	P2 versus P3 = ns
Rumination	3.02 (0.04)	3.61 (0.04)	4.11 (0.06)	P1 versus P2 = ^***^	P1 versus P3 = ^***^	P2 versus P3 = ^***^

P1, “Low SPS - High ER Competency” group; P2, “Moderate SPS – ER Competency” group; P3, “High SPS – Low ER Competency” group; ^*^p < 0.05; ^**^p < 0.01; ^***^p < 0.001; ns, not significant.

Regarding ER strategies, the “High SPS - Low ER Competency” group reported significantly greater use of rumination and lesser use of cognitive reappraisal compared to both the “Low SPS - High ER Competency” group and the “Moderate SPS - ER Competency” group. The “Moderate SPS - ER Competency” group was more likely to employ expressive suppression and rumination than the “Low SPS - High ER Competency” group. Although the “Moderate SPS - ER Competency” group also reported a higher frequency of suppression compared to the “High SPS - Low ER Competency” group, this difference was not statistically significant.

## 4 Discussion

The present study integrated the multidimensional model and the process model to explore how individual differences in SPS impact their ER. Specifically, we employed LPA to identify distinct profiles of SPS and ER competencies among Chinese college students. We then examined the variations in ER patterns across these profiles through the lens of the process model, with a particular emphasis on ER goals and strategies.

The LPA of SPS and five ER competencies resulted in a three-profile solution: “Low SPS - High ER Competency,” “Moderate SPS and ER Competency,” and “High SPS - Low ER Competency.” This categorization partially aligns with our initial hypothesis and resonates with the work of Lionetti et al. (2018), who classified adults’ SPS into high, medium, and low subtypes.

The first profile, labeled “Low SPS - High ER Competency,” was distinguished by having the lowest SPS scores and the highest levels of ER competencies compared to the other two groups. The second profile, “Moderate SPS - ER Competency,” was characterized by intermediate levels of both SPS and ER competencies. The third profile, “High SPS - Low ER Competency,” exhibited the highest SPS scores but the lowest levels of ER competencies. Among the five ER competencies, impulse control emerged as the strongest competency for both the “Low SPS - High ER Competency” group and the “Moderate SPS - ER Competency” group. In the “High SPS - Low ER Competency” group, emotional clarity was identified as the most proficient competency, but it still remained less developed compared to the proficiency levels in other groups. This observation is consistent with our anticipation, based on the studies on alexithymia, that individuals with high SPS are likely to exhibit lower emotional clarity. Despite these varied strengths, engaging in goal-directed behavior was consistently identified as the least developed ER competency across all three profiles.

Compared to female participants, male participants were more likely to be classified into the “Low SPS - High ER Competency” group. This aligns with previous research showing that Chinese females reported higher level of SPS and greater difficulties with ER than males (Wang et al., 2021; Zhang and Zhang, 2023). However, the lack of significant gender differences between the groups with “Moderate SPS - ER Competency” and “High SPS - Low ER Competency” may introduce different insights into the effects of gender on SPS and ER competency. Specifically, the gender impact appears to diminish when males acknowledge their own sensitivity and ER challenges (Lionetti, 2020).

Furthermore, younger participants were more likely to belong to the “High SPS - Low ER Competency” group, whereas no significant age-related differences were found between the “Moderate SPS - ER Competency” group and the “Low SPS - High ER Competency” group. Given that SPS is a temperament trait rooted in biology (Greven et al., 2019), the observed differences across age groups could be due to the older participants’ progressively improved proficiency in ER competencies and their growing adaptability to their sensitivity (Wang et al., 2021). Future research is needed to investigate the developmental changes in the relationship between SPS and ER throughout the lifespan.

Our results revealed significant distinctions in the preferred ER goals across these three groups. In terms of hedonic goals, the “High SPS - Low ER Competency” group demonstrated a marked preference for contra-hedonic goals, a contrast that is especially notable in comparison with the “Low SPS - High ER Competency” group’s inclination towards pro-hedonic goals. Such preferences could be attributed to the fact that SPS consistently exhibited positive correlations with Neuroticism and Introversion (or low Extraversion; Lionetti et al., 2019; Pluess et al., 2023). Previous studies have shown that Extraversion was positively associated with pro-hedonic goals, while Neuroticism was predictive of contra-hedonic goals (Eldesouky and English, 2019a). Furthermore, the inclination towards contra-hedonic goals also indicates that highly sensitive individuals consciously increase or maintain their negative emotions. Considering their enhanced emotional reactivity, this group may favor contra-hedonic goals as a means to authentically engage with and introspectively process their negative emotional experiences.

The finding that both the “High SPS - Low ER Competency” group and the “Moderate SPS - ER Competency” group did not differ significantly in their pursuit of pro-hedonic goals, while also showing less inclination to pursue positive emotions compared to the “Low SPS - High ER Competency” group, offers an intriguing insight. This observation might be explained by a universal inclination towards seeking happiness and positive states, a tendency that extends across varying levels of sensitivity (Tamir and Millgram, 2017). Another explanation could be the unique combination of heightened Neuroticism and Openness associated with high SPS (Lionetti et al., 2019; Pluess et al., 2023). Openness, in particular, has been linked to a predisposition towards seeking pro-hedonic goals among the student sample, according to Eldesouky and English (2019a). Consequently, individuals with higher levels of SPS may be naturally inclined toward both pro-hedonic goals and contra-hedonic goals in ER, driven by their enhanced emotional experiences and strong valuation of both positive and negative experiences (Greven et al., 2019; Eldesouky and English, 2019a). Furthermore, individuals from the “High SPS - Low ER Competence” group and the “Moderate SPS - ER Competence” group are likely to be more engaged in processing both positive and negative stimuli due to their lower ER competency coupled with higher SPS. Such deep engagement demands significant cognitive and emotional resources, which may, in turn, reduce the motivation for actively seeking additional positive experiences. In contrast, individuals with a “Low SPS - High ER Competency” profile, who exhibit reduced emotional reactivity, are not be as reactive to negative emotions as those with other profiles. Consequently, they may demonstrate a more direct pursuit of pro-hedonic goals.

The distinct emphasis on impression management goals by the “High SPS-Low ER Competency” group, compared to the “Moderate SPS - ER Competency” group and the “Low SPS - High ER Competency” group, presents a notable finding. This tendency may be attributed to the increased emotional reactivity typically seen in individuals with high SPS, along with their acute awareness of social nuances (Greven et al., 2019). These characteristics likely heighten their sensitivity to the reactions and expectations of others, leading to a greater likelihood of engaging in impression management goals aimed at gaining approval and avoiding rejection. Furthermore, this inclination for impression management goals in the “High SPS - Low ER Competency” group may provide insight into the established link between high SPS and social anxiety or shyness in adults (e.g., Aron et al., 2005; Hofmann and Bitran, 2007). The emergence of social anxiety or shyness in this population may arise not only from less pleasant early interpersonal interactions (Aron et al., 2005), but also from their intensified concern about how they are perceived by others. Although not significantly different from the “Moderate SPS - ER Competency” group, the “High SPS - Low ER Competency” group appear to be the one that most actively pursues pro-social goals. This preference may be driven by the heightened empathy and deeper emotional connection that sensitive individuals possess (Greven et al., 2019), which further facilitate their relationships with others.

Distinct preferences in ER strategies were evident among these groups. Participants in the “Low SPS - High ER Competency” group predominantly utilized cognitive reappraisal for ER, showing the least tendency to engage in expressive suppression and rumination. Conversely, individuals in the “High SPS - Low ER Competency” group exhibited a significant tendency to employ rumination, while rarely using cognitive reappraisal. This finding partially aligns with previous research, which reported a negative association between SPS and cognitive reappraisal, particularly in the context of negative affect (Eşkisu et al., 2022). Meanwhile, participants with a “Moderate SPS - ER Competency” profile demonstrated a balanced use of both reappraisal and suppression strategies.

The ER habits observed across these groups support the previously established link between ER goals and strategies (Eldesouky and English, 2019b). Specifically, the “Low SPS - High ER Competency” group, predominantly pursuing pro-hedonic goals, demonstrated a preference for cognitive reappraisal. Conversely, the “High SPS - Low ER Competency” group, which focused more on contra-hedonic goals and impression-management goals, exhibited minimal use of cognitive reappraisal. Given the known association between impression management goals and expressive suppression (Eldesouky and English, 2019b; Brandão et al., 2023), the “High SPS - Low ER Competency” group might be expected to predominantly use expressive suppression. However, it was the “Moderate SPS - ER Competency” group that showed a greater tendency towards expressive suppression, an inclination that did not significantly differ from that observed in the “High SPS - Low ER Competency” group.

Two explanations can be considered for this finding. Firstly, individuals with a “High SPS - Low ER Competency” profile may have been raised in environments that failed to equip them with sufficient ER skills, leading to increased rumination, as previously reported by Lionetti et al. (2022). The second explanation concerns the intensity of emotional stimuli perceived by individuals with varying levels of SPS. Specifically, cognitive reappraisal is more likely to be employed when the emotions requiring regulation are perceived as less intense. This strategy has also been shown to be more effective for managing less intense negative emotions. As a result, the “Low SPS - High ER Competency” group, characterized by their reduced emotional reactivity and the proven efficacy of cognitive reappraisal in their life experiences, may be more inclined to employ this strategy. Conversely, for those more sensitive, the emotions they face are typically of higher intensity, making suppression a more appealing option due to its efficacy in managing such emotions (McRae and Gross, 2020).

Overall, the present study investigated the association between SPS and ER among Chinese college students, uncovering patterns consistent with previous findings across different cultural contexts. In contrast to prior research that predominantly focused on the impact of parental attachment on the relationship between SPS and ER, this study instead classified participants based on their SPS and DERS scores. The aim was to delve into the ER processes of highly sensitive individuals, especially those with lower ER proficiency, by focusing on their ER goals and strategies. The findings indicate that individuals with higher SPS and ER deficits tend to favor contra-hedonic goals and impression management goals, and they rely more on response-focused strategies.

This study has several limitations that should be noted. Firstly, the sample predominantly consisted of female college students, which may affect the generalizability of the results to broader populations. For instance, prior research exploring ER goals has identified distinctions between students and community adults (Eldesouky and English, 2019a). Thus, future research with a more diverse range of participants and consideration of cultural differences will be necessary. Secondly, the study only focused on three specific ER strategies, two of which were response-focused. To better reflect the variety of strategies people use in real-life situations, future research should incorporate a wider range of antecedent-focused strategies. Lastly, our reliance on cross-sectional self-report measures presents a limitation. Such measures may not accurately capture the dynamic nature of emotion-related constructs as they fluctuate in daily life. To gain a deeper and more accurate understanding of the relationship between SPS and ER, future research could benefit from employing methodologies such as daily diary methods or experimental designs.

## Data availability statement

The raw data supporting the conclusions of this article will be made available by the authors, without undue reservation.

## Ethics statement

The studies involving humans were approved by the Ethics Committee of the Second Hospital of Shanxi Medical University. The studies were conducted in accordance with the local legislation and institutional requirements. The participants provided their written informed consent to participate in this study.

## Author contributions

YL: Conceptualization, Data curation, Formal analysis, Investigation, Methodology, Writing – original draft. FT: Funding acquisition, Supervision, Writing – review & editing, Methodology.
